# CyclinD1 and p57^kip2^ as biomarkers in differentiation, metastasis and prognosis of gastric cardia adenocarcinoma

**DOI:** 10.18632/oncotarget.18008

**Published:** 2017-05-19

**Authors:** Yi Ru, Xiao-Jie Chen, Zhi-Wei Zhao, Peng-Fei Zhang, Shuai-Hao Feng, Qiang Gao, She-Gan Gao, Xiao-Shan Feng

**Affiliations:** ^1^ The First Affiliated Hospital, and College of Clinical Medicine of Henan University of Science and Technology , Luoyang, Henan 471003, China; ^2^ Medical College, Henan University of Science and Technology , Luoyang, Henan 471003, China; ^3^ Henan Key Laboratory of Cancer Epigenetics, Henan University of Science and Technology , Luoyang, Henan 471003, China; ^4^ Cancer Institute, Henan University of Science and Technology , Luoyang, Henan 471003, China

**Keywords:** gastric cardia adenocarcinoma, p57^kip2^, cyclinD1, clinical stage, different degrees of differentiation

## Abstract

**Objective:**

This study aims to investigate the expression and significance of p57^kip2^ and cyclinD1 in gastric cardia adenocarcinoma (GCA). p57^kip2^ is a negative regulator in the cell cycle. On the contrary, cyclinD1 is a positive regulator of cell cycle progression.

**Methods:**

Thirty-two cases of GCA tissues and adjacent non-cancerous tissues were collected for this study. Immunohistochemistry and fluorescence qualitative PCR was used to determine the level of p57^kip2^ and cyclinD1 in GCA and its adjacent non-cancerous tissues. Furthermore, the correlation between the mRNA/protein and GCA clinical pathologic parameters were analyzed, and the relationship of p57^kip2^ and cyclinD1 in GCA were also evaluated.

**Results:**

The expression of p57^kip2^ significantly lower in GCA (*P* = 0.036), and there was a significant correlation in the different degrees of differentiation (*P* < 0.05). Furthermore, median survival time was 41 months for patients with high mRNA expression of p57^kip2^. This was longer compared to patients with low mRNA expression of P57^kip2^ (37 months, X^2^ = 4.788, *P* = 0.029).The expression of cyclinD1 was significantly higher in GCA(*P* = 0.002), and was significant correlated to clinical stage(P<0.05). Median survival time was 34 months in patients with high mRNA expression of cyclinD1, which was shorter than in patients with low expression of cyclinD1 mRNA (41 months, X^2^ = 4.071, *P* = 0.044). The protein expression of p57^kip2^ was not correlated to the protein expression of cyclinD1 (*P* = 0.55).

**Conclusion:**

The expression of p57^kip2^ and cyclinD1 are likely to suppress or promote the tumorigenesis and progression of GCA.

## INTRODUCTION

In many parts of northern China, gastric cardia adenocarcinoma (GCA) is a common cancer disease [1]. In recent years, studies have continuously revealed that GCA is a malignancy that differs from esophageal malignancy [2]. Furthermore, the regional distribution of GCA is different from gastric and esophageal cancer [3]. Hence, these should be studied as an independent disease. Tumors can be caused by cells that have unlimited autonomous division and proliferation. Normal cell division, proliferation, differentiation and aging maintain the stability of the body. Disorder in cell cycle progression can lead to a chaotic process. The regulation of different checkpoints in cell cycle progression is recognized as the cell cycle regulation mode. The most important checkpoints of the cell cycle are G1/S and G2/M, as well as the junction of the mitotic metaphase/anaphase. An uncontrollable checkpoint would lead to the manifestation of a tumor, and almost all functions of oncogenes and cancer suppressor genes are correlated to cell cycle. As a member of the cyclin-dependent kinases inhibitors (CDKI) and a negative regulatory factor in cell cycle, p57^kip2^ may interfere with cell cycle progression *via* blocking the progression in the G1 phase, exerting an anti-tumor effect [4]. Instead, as a positive regulator of cell cycle progression, cyclinD1 overexpression may accelerate G1/S progression, accelerating cell proliferation [5].

In our study, real-time fluorescence qualitative polymerase chain reaction (PCR), immunohistochemical and western blot assays were employed to detect the expression of p57^kip2^ and cyclinD1 in GCA tissues and its adjacent non-cancerous tissues. The study aimed to investigate the status and significance of p57^kip2^ and cyclinD1 in the occurrence and development of GCA, and explore the potential mechanisms of the inhibitory effect of p57^kip2^ on GCA. Our findings may provide markers for the diagnosis, treatment and prognosis of GCA; which would be beneficial for developing strategies to improve the diagnosis, treatment and prognosis of GCA.

## RESULTS

### MRNA expression of p57^kip2^ and cyclinD1 in GCA and its adjacent non-cancerous tissues

**Figure 1:**
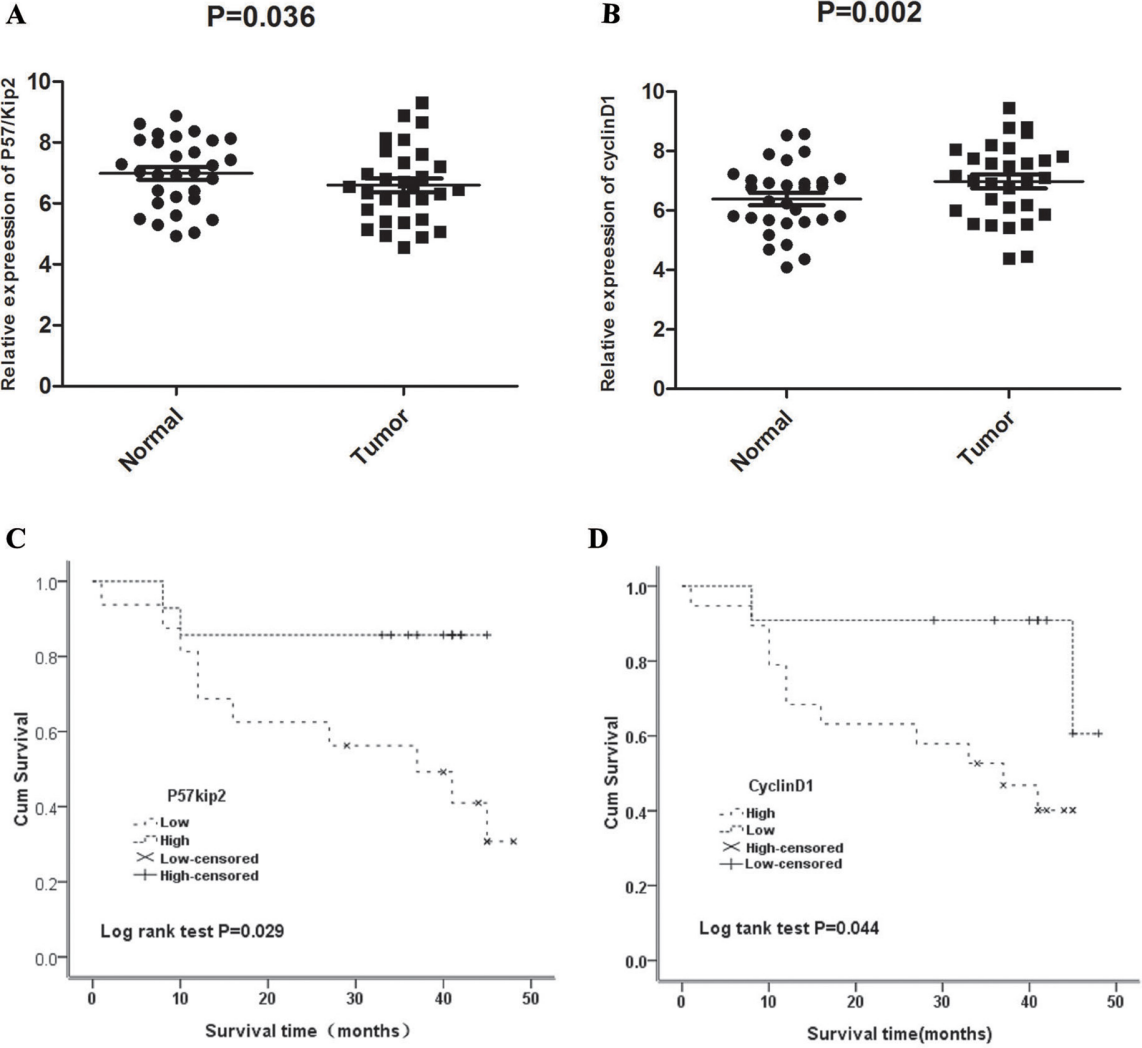
(**A**) Quantitative Real Time PCR(qRT-PCR) of P57/Kip2 mRNA in GCA and the adjacent non-cancerous tissues. (**B**) Quantitative Real Time PCR(qRT-PCR) of cyclinD1 mRNA in GCA and the adjacent non-cancerous tissues. (**C**) Correlation between P57^kip2^ mRNA expression and survival time. (**D**) Correlation between cyclinD1 mRNA expression and survival time.

At mRNA level, the relative expression of p57^kip2^ was 6.985 ± 1.138 and 6.592 ± 1.244 in GCA and its adjacent non-cancerous tissues, respectively; while the relative expression of cyclinD1 was 6.389 ± 1.154 and 6.980 ± 1.286 in GCA and its adjacent non-cancerous tissues, respectively. Significant differences in the expression of p57^kip2^ and cyclinD1 were observed between the two groups (*P* < 0.01 or 0.05, Figure 1).

### Protein expression of p57^kip2^ and cyclinD1 in GCA and its adjacent non-cancerous tissues

**Figure 2:**
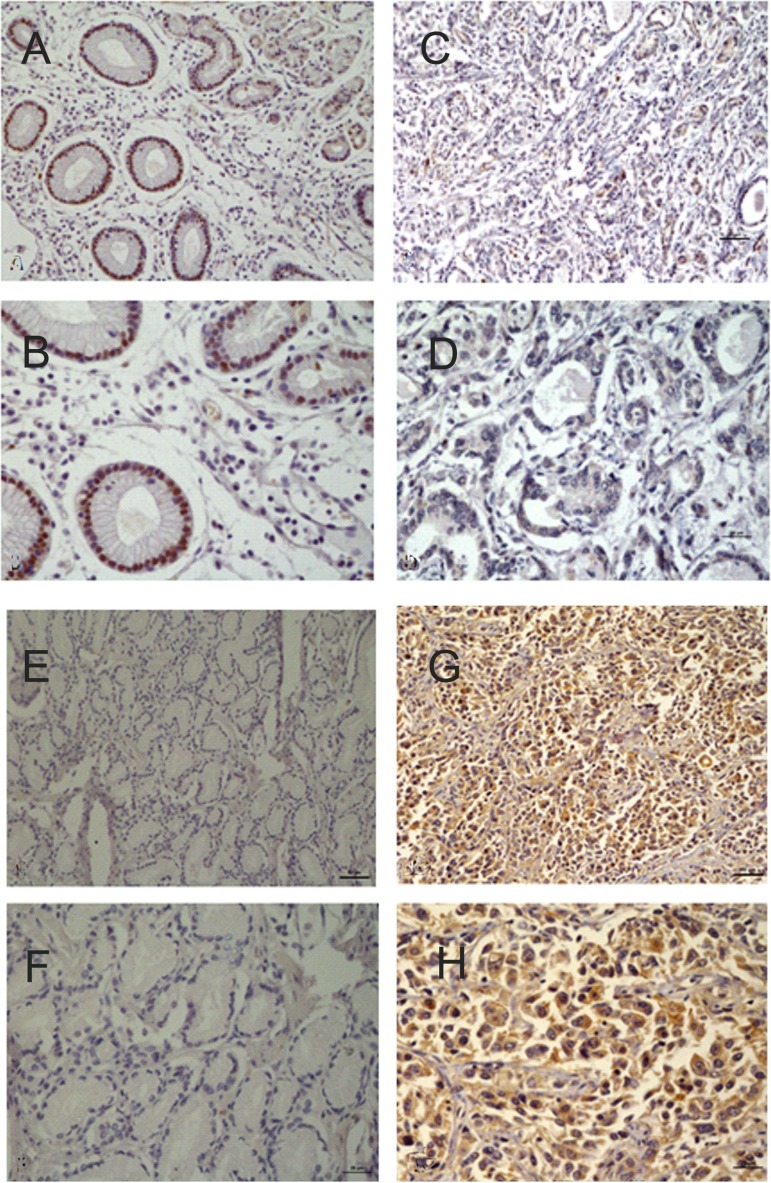
(**A**) Positive expression of P57/Kip2 in the adjacent non-cancerous tissues (×20). (**B**) Positive expression of P57/Kip2 in the adjacent non-cancerous tissues(×40). (**C**) Negative expression of P57/Kip2 in GCA (×20). (**D**) Negative expression of P57/Kip2 in GCA(×40). (**E**) Negative expression of CyclinD1 in the adjacent non-cancerous tissues(×20). (**F**) Negative expression of CyclinD1 in the adjacent non-cancerous tissues(×40). (**G**) Positive expression of CyclinD1 in GCA(×20). (**H**) Positive expression of CyclinD1 in GCA(×40).

At protein level (immunohistochemistry), 6 and 26 patients were positive for p57^kip2^ in GCA and its adjacent non-cancerous tissues, respectively; while 27 and 5 patients were positive for cyclinD1 in GCA and its adjacent non-cancerous tissues, respectively. Significant differences in p57^kip2^ and cyclinD1 expression were observed between the two groups (*P* < 0.05 or0.01, Figure 2).

**Figure 3:**
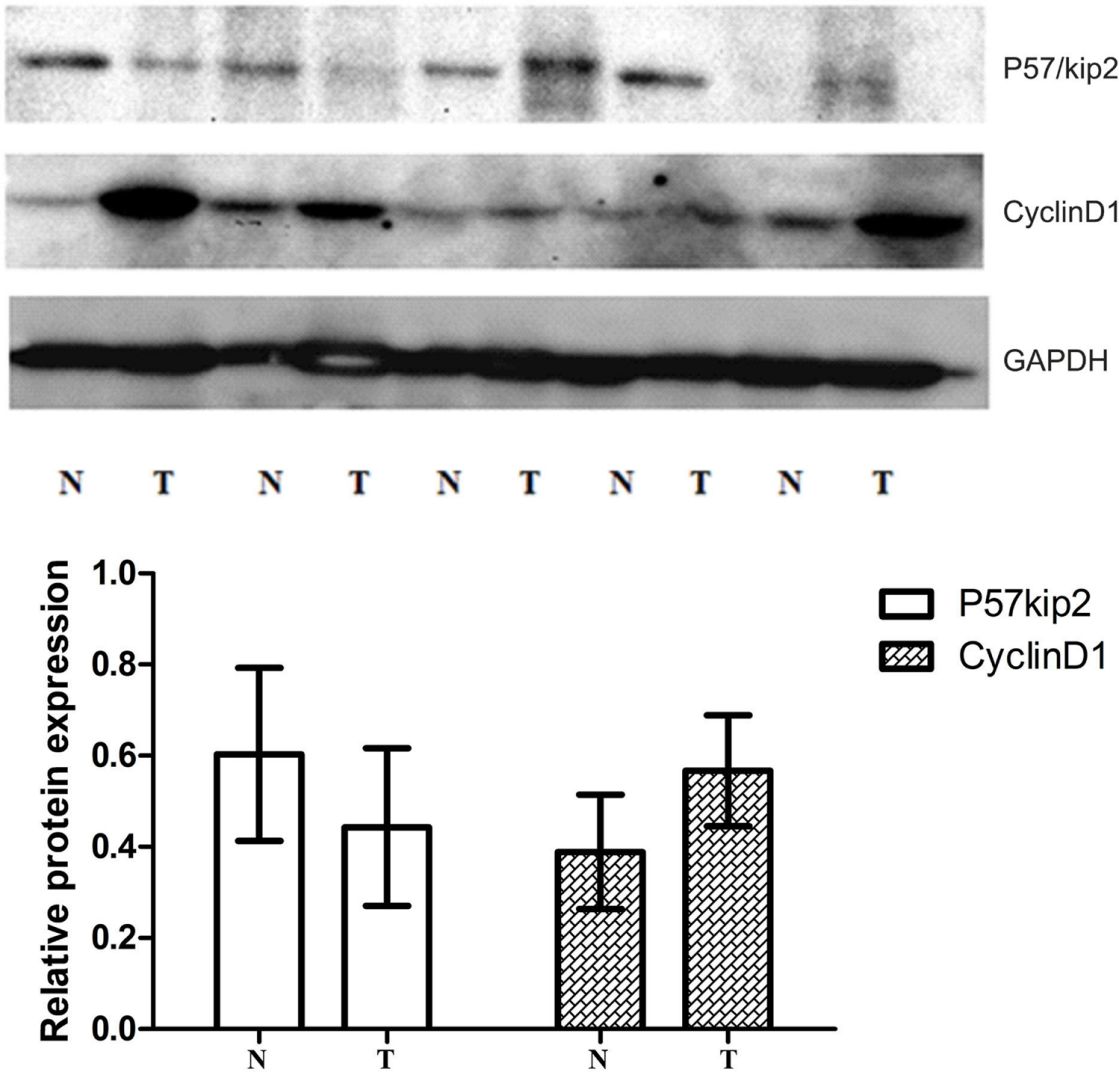
Western-blot (WB) of tissue cycclinD1 protein and P57/Kip2 protein in GCA and the adjacent non-cancerous tissues

At protein level (western blot), the relative expression of p57^kip2^ was 0.414 ± 0.170 and 0.601 ± 0.218 in GCA and its adjacent non-cancerous tissues, respectively; while the relative expression of cyclinD1 was 0.587 ± 0.112 and 0.386 ± 0.109 in GCA and its adjacent non-cancerous tissues, respectively. Significant differences in the expression of p57^kip2^ and cyclinD1 were observed between the two groups (*P* < 0.05or 0.01, Figure 3).

### The correlation of p57^kip2^ and cyclinD1 mRNA expression with the clinicopathological features of GCA

Based on the expression levels of p57^kip2^ mRNA obtained by qRT–PCR, we divided the 32 GCA patients into a high- p57^kip2^ expression group (*n* = 18) and a low- p57^kip2^ expression group (*n* = 14) according to the median expression of tumor tissue (6.514). In the same way , we divided patients into a high- cyclinD1 expression group (*n* = 21) and a low- cyclinD1expression group (*n* = 11) according to the median expression of tumor tissue (7.022). Then we analyzed the correlation of p57^kip2^ and cyclinD1 expression levels with the clinicopathological characteristics of patients with GCA (Table 1). Significant differences were observed in p57^kip2^ mRNA expression among patients with different degrees of differentiation, and cyclinD1 among patients with different clinical stages (*P* < 0.01 or 0.05). In addition, patients with lymph node metastasis had different mRNA expression levels of cyclinD1, compared with patients without lymph node metastasis. Moreover, there were no differences in p57^kip2^ and cyclinD1 mRNA expression between male and female patients, among patients with or without stomach diseases, among patients with or without a family history of cancer, and among patients in different age groups (*P* > 0.05, Table 1).

**Table 1:** **Correlation of mRNA expressions of p57**^Kip2^ and CyclinD1 with clinicopathological features of GCA

Clinicopathological features	*n*	Relative expression of CyclinD1	*P*	Relative expression of p57/Kip2(%)	*P*
Age (year)			0.320		0.586
< 60	11	6.825 ± 1.121		6.456 ± 1.031	
≥ 60	21	6.995 ± 1.157		6.359 ± 1.157	
Male/female			0.218		0.353
Male	30	7.121 ± 1.058		6.268 ± 1.227	
Female	2	6.877 ± 1.203		6.448 ± 1.090	
The stomach diseases			0.307		0.310
No	12	6.827 ± 1.214		6.516 ± 1.012	
Yes	20	6.993 ± 1.124		6.209 ± 1.272	
Family history of cancer			0.445		0.162
No	19	6.895 ± 1.212		6.451 ± 1.147	
Yes	13	6.996 ± 1.130		6.173 ± 1.248	
Pathological grade differentiated			0.312		0.032
Middle-low differentiated	7	6.988 ± 1.133		6.083 ± 1.215	
High-middle differentiated	25	6.810 ± 1.209		6.513 ± 1.012	
Clinical stage			0.021		0.697
< III_b_	15	6.620 ± 1.214		6.408 ± 0.127	
≥ III_b_	17	7.283 ± 1.131		6.363 ± 0.138	
Lymph node metastasis			0.042		0.068
Negative	8	6.493 ± 1.169		6.521 ± 1.009	
Positive	24	6.876 ± 1.140		6.091 ± 1.185	

### The correlation of p57^kip2^ and cyclinD1 protein expression with the clinicopathological features of GCA

Significant differences were observed in the protein expression of p57^kip2^ among patients with different degrees of differentiation, and in the protein expression of cyclinD1 among patients with different clinical stages (*P* < 0.01 or 0.05). There were no differences in the protein expression of p57^kip2^ and cyclinD1 between genders, among patients with or without stomach diseases, among patients with or without a family history of cancer, and patients in different age groups (*P* > 0.05, Table 2).

**Table 2:** Correlation of protein expressions of p57^Kip2^ and CyclinD1 with clinicopathological features of GCA

Clinicopathological features	CyclinD1 positive (%)	CyclinD1 negative (%)	*P*	p57Kip2 negative (%)	p57Kip2 positive (%)	*P*
Age (year)			0.773			0.637
< 60	2(40.0)	9(33.3)		10(38.5)	1(16.7)	
≥ 60	3(60.0)	18(66.7)		16(61.5)	5(83.3)	
Male/female			0.292			0.345
Male	4(80.0)	26(96.3)		25(96.2)	5(83.3)	
Female	1(20)	1(3.7)		1(3.8)	1(16.7)	
The stomach diseases			0.053			0.370
No	4(80.0)	8(29.6)		11(42.3)	1(16.7)	
Yes	1(20.0)	19(70.4)		15(11.5)	5(11.5)	
Family history of cancer			0.645			0.185
No	3(60.0)	16(44.4)		14(53.8)	5(83.3)	
Yes	2(40.0)	11(55.6)		12(46.2)	1(16.7)	
Pathological grade differentiated			1.000			0.012
Middle-low differentiated	1(20.0)	6(22.2)		3(11.5)	4(66.7)	
High-middle differentiated	4(80.0)	21(77.8)		23(88.5)	2(33.3)	
Clinical stage			0.015			0.637
< III_b_	5v100)	10(37.0)		10(38.5)	1(16.7)	
≥ III_b_	0(0)	17(63.0)		16(61.5)	5(83.3)	
Lymph node metastasis			0.578			0.117
Negative	2(40.0)	6(22.2)		8(30.8)	0(0)	
Positive	3(60.0)	21(80.0)		18(69.2)	6(100)	

### The correlation between p57^kip2^ and cyclinD1 protein expression in GCA

On the basis of the median expression of p57^kip2^ and cyclinD1 in GCA, patients were divided into four groups; and the relation between the expression of p57^kip2^ and cyclinD1 protein was assessed. We found that p57^kip2^ protein expression was not correlated to cyclinD1 protein expression (r = –0.202, *P* = 0.55, Table 3).

**Table 3:** Correlation between p57/Kip protein expression and CyclinD1 protein expression in GCA

p57Kip2	CyclinD1	
	+	–
+ –	6 21	0 5

*P* = 0.55.

### Correlation of the mRNA expression of p57^kip2^ and cyclinD1 with the prognosis of GCA

Median survival time was 41 months in GCA patients with high mRNA expression of p57kip2, which was longer than in patients with low mRNA expression of p57kip2 (37 months, X2 = 4.788, *P* = 0.029; Figure 1C). Furthermore, median survival time was 34 months in patients with high mRNA expression of cyclinD1, which was shorter than in patients with low mRNA expression of cyclinD1 (41 months, X2 = 4.071, *P* = 0.044; Figure 1D). A univariate Cox analysis showed that the stomach diseases, lymph node metastasis, TNM stage, CyclinD1 and p57Kip2 expression were correlated with the survival (Table 4). Multivariate analysis using the Cox proportional hazard model demonstrated that CyclinD1 and p57Kip2 expression was an independent risk factors for OS (*P* = 0.027, *P* = 0.008,Table 4) in addition to TNM stage (*P* = 0.000, Table 4). These results identified that overexpression of CyclinD1 or lower expression of p57Kip2 seemed to be a predictive factor of poor survival of GCA, suggesting that they may contribute to GCA pathogenesis and can be employed as powerful independent prognostic factors.

**Table 4:** Univariate and multivariate analysis of different prognostic factors for OS in 32 patients with GCA

	Univariate analysis			Multivariate analysis		
Prognostic factors	HR	95% CI	*P* value	HR	95% CI	*P* value
Age (> 60/≤ 60)	0.662	0.313–1.126	0.253			
Gender (Male/Female)	1.122	0.614–2.179	0.799			
The stomach diseases (NO/YES)	1.822	1.135–3.026	0.039*			
Family history of cancer (NO/YES)	0.821	0.603–1.192	0.281			
Differentiation (Poor/Well+Moderate)	1.892	1.702–3.148	0.017*			
lymph node metastasis (Presence/Absence)	4.246	1.895–6.854	0.000*			
TNM stage (I+II/III+IV)	7.247	3.072–9.100	0.000*	4.972	2.133–6.376	0.000*
CyclinD1 (High/Low)	2.383	1.302–4.361	0.005*	2.019	2.682–6.378	0.027*
p57Kip2 (High/Low)	0.172	0.032–0.461	0.003*	0.169	0.039–0.489	0.008*

TNM, tumour-node-metastasis staging system; HR, hazard ratio; CI, confidence interval.*P < 0.05.

## DISCUSSION

Cancer is an outcome of the cooperation and interaction of multi-factors, and its progression involves multiple genes and multiple steps. This is true for GCA. Recurrence and metastasis are major problematic issues that threaten human health. More investigators continue to propose that conjoint multi-markers analysis would be superior to one marker in predicting the cancers prognosis. With the development of biotechnology, increasing genes that may promote or inhibit cancer development have been identified. Therefore, exploring the correlation between genes that play a role in inhibiting or promoting cancer development is important for predicting the prognosis and treatment efficacy of cancers in clinic.

P57^kip2^ is a tumor metastasis suppressor. The deficiency or downregulation of p57^kip2^ expression have been observed in multiple cancers including lymphomas [7], gastric cancer [8], pancreatic cancer [9], breast cancer [10], bladder cancer [11], and prostate cancer [12]. In most tumors, the main means that lead p57^kip2^ to inactivate were reported as modifications after transcription and translation. The lack and loss of heterozygosity, DNA methylation and histone acetylation, microRNAs, many signal transduction pathways and phosphorylation, and ubiquitination in the regulation of p57^kip2^ expression promote the malignant biological behavior of cancer cells [13–17]. The inhibitory activity of cyclin/CDK depends on the amino terminal domain and the nuclear localization signal (NLS) at the carboxy terminal, they were CDK binding/inhibitory region. In contrast, CIP/KIP proteins exhibit divergent carboxy terminal domains. Human p57^kip2^ and p21CIP1 has a proliferating cell nuclear antigen (PCNA) binding domain required for inhibiting DNA replication [18]. What's more, extracellular factors such as TGF-β, IGF-1 and pituitary adenylate cyclase-activating peptide [19–21], and transcription factors such as CTIP2 and HES1 [22, 23], which are known to play important roles during corticogenesis, regulate the positive or negative expression of p57^kip2^. Shin revealed that p57 serves as a tumor suppressor in GCA [8]. However, the functional role of p57 in GCA remains unclear. Our results determined that p57^kip2^ can serve as a tumor suppressor in GCA. Furthermore, our results revealed that the mRNA and protein expression of p57^kip2^ were consistent. The p57^kip2^ expression in GCA was markedly lower than in adjacent non-cancerous tissues. In addition, p57^kip2^ expression was different in patients with different degrees of differentiation. That is, the expression of p57^kip2^ in patients with poorly differentiated GCA was significantly lower than in patients with well-differentiated GCA. These findings suggest that the more invasive capabilities are, the lower the p57^kip2^ expression becomes. However, there was no marked difference in p57^kip2^ expression between genders, patients in different age groups and in different clinical stages. Furthermore, there was no marked difference among patients with or without stomach diseases, and patients with or without a family history of cancer. The survival curve analysis revealed that patients with high mRNA expression of p57^kip2^ had a median survival time of 41 months, which was longer than in patients (34 months) with high mRNA expression of p57^kip2^. (*X*^2^ = 4.788, *P* = 0.029).

The cyclinD1 gene is located in 11q13 and possesses five exons and four introns. Its length is approximately 15 kb, and encodes 295 amino acids with a molecular weight of 34 kD. CyclinD1 expression undergoes periodic alteration with the progression of the cell cycle under physiological conditions. Generally, cyclinD1 is synthesized in the G0 phase. Its complex reaches a maximal level in the G1 phase and is reduced in the S phase. CyclinD1 expression remains at a low level in other phases of the cell cycle. Furthermore, cyclinD1 can promote the progression of the cell cycle and regulate cell proliferation. Evidence has shown that cyclinD1 acts in a retinoblastoma (Rb)-dependent manner [24]. The over-expression of cyclinD1 may cause the transition across the G1/S checkpoint, influence the normal regulation of the cell cycle, and result in rapid cell proliferation; which finally causes the formation of cancers. There are three members in cyclinD family: cyclinD1, cyclinD2 and cyclinD3. The overexpression of cyclinD1 can be detected in breast cancer [25], as well as in head and neck carcinoma [24]. It is attributed to the promotion of cell cycle progression of cyclinD1. CyclinD1 expression is very low in normal breast tissues. In breast cancer, the mRNA of cyclinD1 is 25-81% and protein expression is 45-83%, respectively [26, 27]. Umekita *et a*l. [28] followed-up 173 patients with breast cancer after surgery, and their results revealed that the expression of cyclinD1 was at a high level in most patients, and that this could be used as an independent factor for indicating the prognosis of ER negative breast expression. In breast cancer, Wang *et al.* [29] investigated the expression of cyclinD1 in the metastasis and migration of breast cancer. The study revealed that the expression of cyclinD1 was correlated to the metastasis of breast cancer, and breast cancer with high cyclinD1 expression had a poor prognosis. The results suggest that cyclinD1 may facilitate the migration of cancer cells and promote their growth. Furthermore, cyclinD1 could effect on cell proliferation not only at the cellular level, but also *in vivo* (such as the ESCC animal model [30]). In ESCC, the increased expression of cyclinD1 was related to a poor prognosis [31]. Cattani *et al.* [32] found that in laryngeal cancer, cyclinD1 expression was associated with HPV infection. CyclinD1 expression was at a high level in patients with HPVE6/7 infection, and interfered with the cell cycle; resulting in the occurrence of laryngeal cancer. Our results revealed that the mRNA and protein expression of cyclinD1 were consistent in GCA. The expression of cyclinD1 In GCA was markedly higher than in its adjacent non-cancerous tissues. In addition, cyclinD1 expression was different in patients with different clinical stages and in patients with and without metastasis. That is, the expression of cyclinD1 in patients with clinical stage III_b_-IV GCA was significantly higher than in patients with clinical stage I- IIIa GCA. These findings suggest that the higher the cyclinD1 expression, the more potent the metastatic and invasive capability of cancer cell in GCA is. However, there is no marked difference in cyclinD1 expression between genders, among patients in different age groups, and between patients with and without gastric diseases. Survival curve analysis revealed that patients with low cyclinD1 mRNA expression had a median survival time of 41 months, which was longer than in patients (34 months) with high cyclinD1 mRNA expression (*X*^2^= 4.071, *P* = 0.044).

## MATERIALS AND METHODS

### Sample collection

Between January 2012 and March 2014, 32 patients with GCA were recruited from the Department of Thoracic Surgery in the First Affiliated Hospital of Henan University of Science and Technology. These patients received operative treatment, and GCA was pathologically confirmed. All patients did not receive radiotherapy and chemotherapy before surgery, and had complete clinical, pathological and follow-up data. Among these patients, 30 patients were male and two patients were female; and the median age of these patients was 61.4 years (range: 39-76 years). GCA was classified as highly or moderately differentiated GCA (*n* = 25), and middle-low differentiated GCA (*n* = 7). Clinical pathologic staging was performed according to the TNM staging system formulated by the Union for International Cancer Control (UICC, 1997). Clinical staging was performed as a stage < IIIB GCA (*n* = 15) and as tage ≥ IIIB (*n* = 17). Additionally, 24 patients were diagnosed with lymph node metastasis, and eight patients had no lymph node metastasis. The follow up period ranged between 1–48 months. The GCA and its adjacent non-cancerous tissues were collected, divided into two groups, and stored in liquid nitrogen.

### Materials and reagent

Total RNA isolation kit (Shanghai Tiangen Biotech Co., Ltd.), qPCR Master Mix kit(Vazyme Biotech Co., Ltd.), reverse transcription kit (Vazyme Biotech Co., Ltd.), antibodies against p57^kip2^ and cyclinD1 (Abcam Biotech Co., Ltd., UK) and primers (Table 5, Shanghai Sangon) were used in this study.

**Table 5:** **Primers for P57**^kip2^, CyclinD1 and β-actin Gene Sequence Length (bp)

Gene	Sequence	Length(bp)
P57kip2	5′- CCACCTAGCTTGCAGTCTCTT -3′ 5′- TACAGTCGGCTCAGGAACCA -3′	83
CyclinD1	5′- GATGAGGAAGAGTTGCTAGAAGAG -3′ 5′- TCGTCAGCCAATCGGTAGTAG -3′	163
β-actin	5′-CCC AGC ACA ATG AAG ATC AAG ATC AT-3′ 5′-ATC TGC TGG AAG GTG GAC AGC GA-3′	101

### Detection of p57^kip2^ and cyclinD1 mRNA expression(qPCR)

Total RNA was extracted by TRIZOL Regent kit. UV spectrophotometer was used to determine the concentration of RNA. The first strand of RNA was synthesized with reverse transcription. Fluorescence qualitative PCR was performed in 20-μl mixture (Amplification primers are shown in Table 5), and the conditions as follows: denaturation at 95°C for five minutes, 95°C for 10 seconds, annealing at 60°C (58°C for β-actin, 56°C for p57^kip2^ and cyclinD1), and extension at 72°C for 20 seconds for 40 cycles. Three wells were used for each group. The 2^-∆∆CT^ method was used to calculate the expression of p57^kip2^ and cyclinD1.

### The detection of p57^kip2^ and cyclinD1 protein expression

*Western blot* Tissues were kept in liquid nitrogen and total protein was extracted with RIPA lysis buffer. The BCA method was used to determine the protein concentration. The protein solution was mixed with a loading buffer, and boiled for use. Then, 50 μg of total protein were loaded for electrophoresis at 20 mA for three hours, and transfer membrane was performed at a constant current for two hours. Next, the membrane was blocked with 5% non-fat milk at room temperature for one hour, and treated with the primary antibody at a ratio of 1:1,000, overnight at 4°C. After washing three times with TBST (for 10 minutes each), the membrane was treated with HRP conjugated secondary antibody at a ratio of 1:3,000 for two hours at room temperature. Then, the membrane was washed three times with TBST (for 10 minutes each), and visualization was performed using an ECL kit. β-actin was used as an internal control.

### Immunohistochemical analysis

Sample slices for pathologic examination were reviewed to select the appropriate areas of GCA and its adjacent non-cancerous tissues. Tissue blocks that were matched for representative GCA and its adjacent non-cancerous tissues were collected from the tissue bank for the immunohistochemical assay of p57^kip2^ and cyclinD1. Tissue blocks to tissues from peripheral areas next to the tumor margin were standardized for all cases. Immunohistochemical analysis of the slices was performed by indirect immunoperoxidase staining [6]. The tissue specimens were incubated with diluted primary antibodies (1:250 dilution for p57^kip2^ and 1:400 dilution for cyclinD1), and control specimens were treated with PBS. Result analysis of slices for immunohistochemistry was performed by two independent pathologists, who had no prior knowledge on the data of the patients. The pathologists measured the immunohistochemical activities separately. Immunohistochemical staining was measured semi-quantitatively on the basis of the depth of staining, and were classified as follows: negative, when the average proportion of positive cells in 10 visual fields was < 25% under a 40× microscope; positive, when the average proportion of positive cells in 10 visual fields was > 25% under a 40× microscope.

### Statistical analysis

Statistical analysis was performed using SPSS version 17.0 for Windows. Comparisons between the two groups were performed with *t-test*. Qualitative data (the expression of p57^kip2^ and cyclinD1) were analyzed using Chi-square test. Pearson analysis was used to calculate the correlation between clinicopathological features and the expression of p57^kip2^ and cyclinD1. The survival curve was calculated using the Kaplan-Meier method. Survival time was evaluated using the log-rank test. Univariate analysis and multivariate models were fit using a Cox proportional hazards regression model. A *P*-value < 0.05 was considered statistically significant.

## CONCLUSIONS

In the present study, we also evaluated the relationship between the protein expression of p57^kip2^ and cyclinD1. Results revealed that the protein expression of p57^kip2^ was negatively correlated to the protein expression of cyclinD1 (*P* = 0.55). In GCA and its adjacent non-cancerous tissues, the expression of P57^kip2^ and cyclinD1 were significantly different; which implies that both proteins are involve in the malignant transformation of benign tissues, and both might exert a facilitation effect in this process. The relationship between these two proteins needs to be elucidated in future studies. Our findings indicate that both p57^kip2^ and cyclinD1 may become promising markers for predicting the prognosis of GCA, which is beneficial for the diagnosis and treatment of GCA.

## Abbreviations

GCA: Gastric cardia adenocarcinoma
PCR: Polymerase chain reaction
RNA: Ribonucleic Acid
CDKI: Cyclin-dependent kinases inhibitors
RIPA: Radio Immunoprecipitation Assay
TBST: Tris-Buffered Saline and Tween 20
HRP: Horseradish Peroxidase
CTIP2: Chicken ovalbumin upstream promoter transcription factor-interacting protein 2
HES1: Hairy enhancer of split
ESCC: Esophageal squamous cell carcinoma
HPV: Human papilloma virus
